# A Bibliometric Analysis of the 100 Most-Cited Articles on Soccer Injuries

**DOI:** 10.7759/cureus.82636

**Published:** 2025-04-20

**Authors:** Amr Chaabeni, Meryam M Slama, Amine Kalai, Helmi Ben Saad, Alejandro López-Valenciano, Zohra Ben Salah Frih, Anis Jellad

**Affiliations:** 1 Physical Medicine and Rehabilitation Department, University of Monastir, Faculty of Medicine, Monastir, TUN; 2 Service of Physiology and Functional Explorations, University of Sousse, Farhat Hached University Hospital, Sousse, TUN; 3 Physical Exercise and Performance Research Group, Department of Education Science, School of Humanities and Communication Sciences, Universidad Cardenal Herrera-CEU, Castellón de la Plana, ESP; 4 Laboratory of Technology and Medical Imaging-LR12ES06, Center for Musculoskeletal Biomechanics Research, Faculty of Medicine, University of Monastir, Monastir, TUN

**Keywords:** bibliometric research, football injury, injury epidemiology, prevention, soccer, trend analysis

## Abstract

Bibliometric analysis is increasingly utilized to assess specific research fields and identify emerging areas of interest. While it is commonly employed in sports medicine research to assess injuries, few studies have concentrated on soccer. The aim of this study was to identify the top 100 most-cited publications related to soccer injuries and conduct a comprehensive bibliometric mapping analysis to understand research trends. The bibliographic search was performed on November 16, 2022, using the Clarivate Analytics Web of Science Database, and the 100 most-cited articles related to soccer injuries were identified and reviewed. TheBibliometrix R-package software ( https://www.bibliometrix.org/) was used for data analysis. The 100 most-cited articles were published between 1990 and 2017. The majority of articles were published in the British Journal of Sports Medicine (32 articles) and the American Journal of Sports Medicine (31 articles). Most corresponding authors (26%) originated from Sweden. Approximately half of the articles were observational studies using level 2 evidence. The primary research topics included epidemiology (25 articles) and prevention (24 articles). Most articles focused on male (53%), adult (57%), and elite soccer players (51%), while the majority of articles about female players (76.9%) ranked in the top 50 cited articles. This study underscores the necessity for more comprehensive research on soccer injuries, with a particular focus on female players, to bridge existing knowledge gaps and enhance injury prevention strategies.

## Introduction and background

Introduction

Soccer is the most widely played sport in the world, with more than 265 million participants [1,2]. However, its practice is associated with a high rate of injuries, with an overall incidence of 8.1 injuries per 1,000 hours of exposure [3]. It is estimated that professional soccer teams experience an average of two injuries per player in a single season [4]. As a result, professional teams and athletes may face significant challenges related to practice time loss and financial burdens [5]. This may explain many authors’ growing interest in the study of soccer injuries.

Bibliometric studies are defined as a statistical assessment of published research aiming to quantify the impact and analyze the trends of publications, especially those with a high number of citations [6-8]. Bibliometric analysis involves evaluating citation patterns, publication trends, and author collaborations, enabling researchers to identify key contributors and emerging areas of study. Thus, bibliometric studies offer a great opportunity for researchers to identify the most relevant and influential papers in their specific field and to construct an opinion about the progression of ideas related to epidemiological, treatment, and preventive trends [6].

In the context of sports medicine, bibliometric studies provide valuable insights into how research on soccer injuries has evolved and help to pinpoint gaps in the literature. For research focused on soccer, bibliometric studies have focused on general topics, such as medicine and science in football [9,10]. We aim to offer a valuable resource for researchers, practitioners, and stakeholders in soccer player health and performance. We anticipate outcomes that will establish a foundation for understanding main trends, provide crucial insights into preventive measures, and offer an informed basis for future research directions in reducing the impact of injuries in soccer. To the best of the authors’ knowledge, no previous bibliometric studies have dealt with soccer injuries. The purposes of the present bibliometric study were to identify the top 100 most-cited articles related to soccer injuries, and to carry out a thorough mapping analysis. This article was previously posted to the medRxiv preprint server on April 28, 2023 (https://www.medrxiv.org/content/10.1101/2023.04.27.23289221v1) and updated in this version.

## Review

Search strategy

We researched the Web of Science (WoS) database using Clarivate Analytics on November 16, 2022. The search terms used were as follows: Topic Sentence (soccer OR football) NOT (American football OR Australian football OR rugby OR hockey OR cricket). There were no restrictions related to the type of study, the language, or publication date.

**Figure 1:**
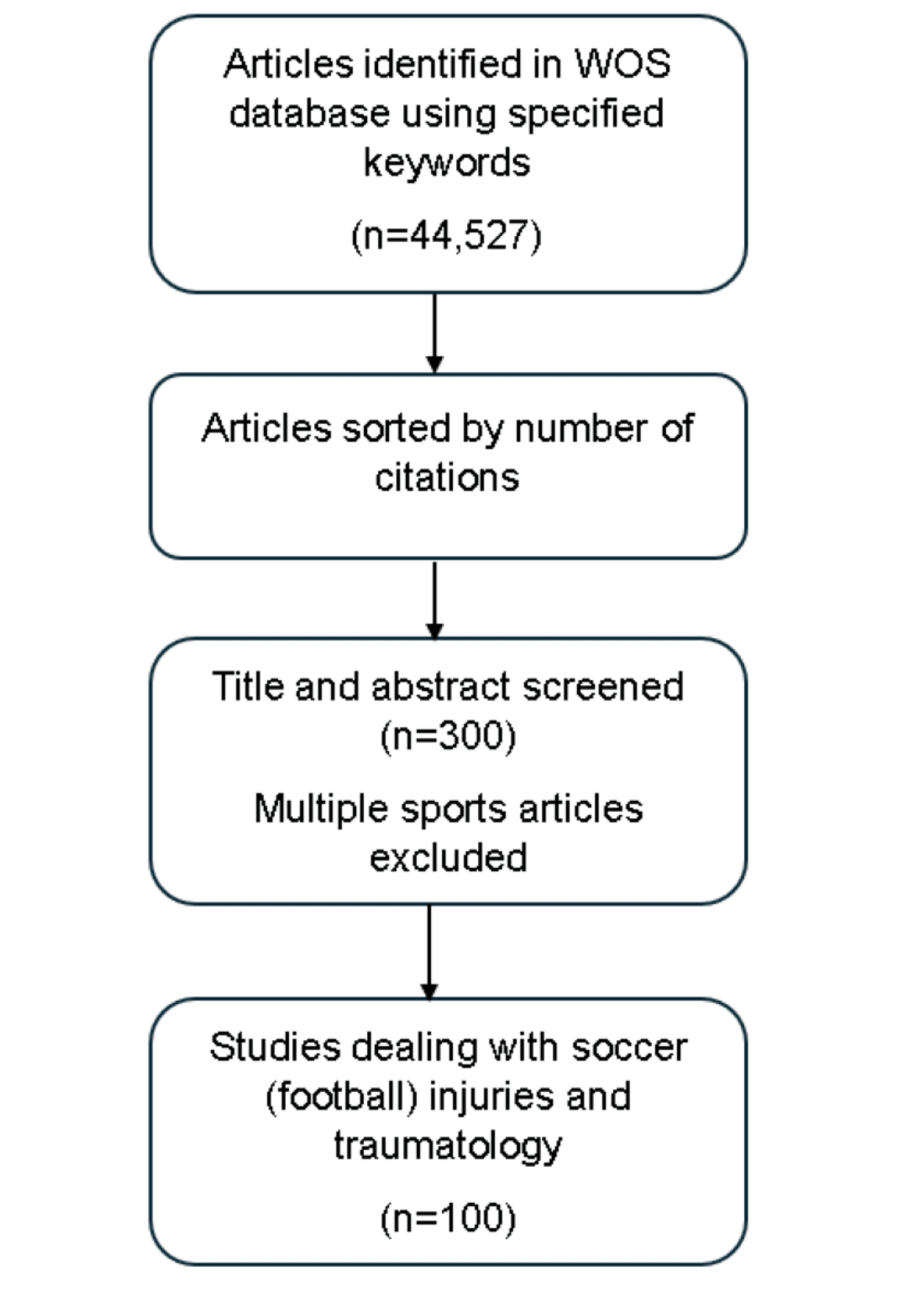
Study selection flowchart WoS: Web of Science.

Using the specified search terms, we found 44,527 articles on the WoS database. Articles were sorted by the number of citations. Three authors (AC, MS and AK in the authors’ list) assessed the first 300 most-cited articles; the reviewers individually verified the title and abstract of each article and kept only those dealing with injuries and traumatology that were related to soccer (football) athletes. Articles comprising multiple sports were excluded. The analytical study was limited to the 100 most-cited articles (Figure 1).

Data analysis

Bibliometrix R-package software ( https://www.bibliometrix.org/) was used for data analysis. The resulting 100 most-cited articles were analysed to obtain the following variables: journal title, authors’ names, authors’ country of origin, year of publication, number of citations, citations per year, article type (eg; original research, letters, review articles, case studies, and methodologies or methods) [11], study design (eg; descriptive studies, observational studies, experimental studies, reviews, and methodologies) [12], and level of evidence according to the standards set by the Journal of Bone and Joint Surgery, which involves ranking articles based on the study design employed to address the primary research question [13,14]. Authors’ keywords were extracted to identify the trends [15].

Each article was assigned to topics based on the research question the authors attempted to address, such as epidemiology, anatomy and biomechanics, prevention, rehabilitation, classification and scoring, imaging, injury mechanism, surgical technique, and outcome, and neuropsychology. Additionally, demographic information about the athletes, such as sex (e.g., male, female, or both), competition level (e.g., elite, non-elite, or both), and age category (e.g., youth under 18, adults over 18) was obtained from the original research articles. For the competition level, professional players or those playing in the first division were classified as “elite”, while amateur, college, or high-school players were classified as “non-elite” [9].

Results

The top 100 most-cited articles are listed in the appendix with their rank, number of citations and citations per year. These articles had a total of 26046 citations, with an average (range) of 260.5 (146-945) citations per article. Table 1 exposes the distribution of journals per citations and articles. The top 100 most-cited articles were published in 21 journals. The two journals that published the most articles were the British Journal of Sports Medicine (n=32 articles, 8425 citations) and the American Journal of Sports Medicine (n=31 articles, 8119 citations).

**Table 1:** Top 100 most-cited articles on soccer injuries The table shows journals distribution per total citations, number of articles, and year of publication.

Journals	Total citations	Number of articles	Year of publication
British Journal of Sports Medicine	8425	32	2001
American Journal of Sports Medicine	8119	31	1991
Scandinavian Journal of Medicine & Science in Sports	2175	9	1996
Knee Surgery Sports Traumatology Arthroscopy	1471	6	2000
Arthritis and Rheumatism	945	1	2004
British Medical Journal	673	2	2008
Journal of Athletic Training	610	3	2007
Sports Medicine	585	3	1994
Annals of The Rheumatic Diseases	557	1	2004
JAMA-Journal of The American Medical Association	433	2	1999
Lancet	273	1	1999
Clinical Journal of Sport Medicine	246	1	2006
Journal of Orthopaedic & Sports Physical Therapy	244	1	2010
BMJ-British Medical Journal	228	1	2012
Radiology	226	1	2013
Neurology	216	1	1998
Journal of Pediatric Orthopaedics	189	1	2004
Foot & Ankle	184	1	1990
Acta Orthopaedica Scandinavica	162	1	1995
Journal of Science And Medicine in Sport	159	1	2010
Medicine and Science in Sports And Exercise	154	1	2003

The number of authors who contributed to the top 100 most-cited articles was 279. Table 2 lists the authors with five or more articles. The top three authors were Martin Hagglund (Hagglund M, n=20 articles), Jan Ekstrand (Ekstrand J, n=19 articles), and Markus Walden (Walden M, n=16 articles).

**Table 2:** List of authors with five or more articles

Authors	Total citations	Number of articles	Publishing year start
Martin Hagglund	6208	20	2005
Jan Ekstrand	5976	19	1990
Martin Walden	4764	16	2005
Astrid Junge	2520	10	2000
Thor Einar Andersen	2271	6	2006
Jiri Dvorak	2118	11	2000
Ingar Holme	1742	5	2004
Roald Bahr	1729	5	2004

**Figure 2:**
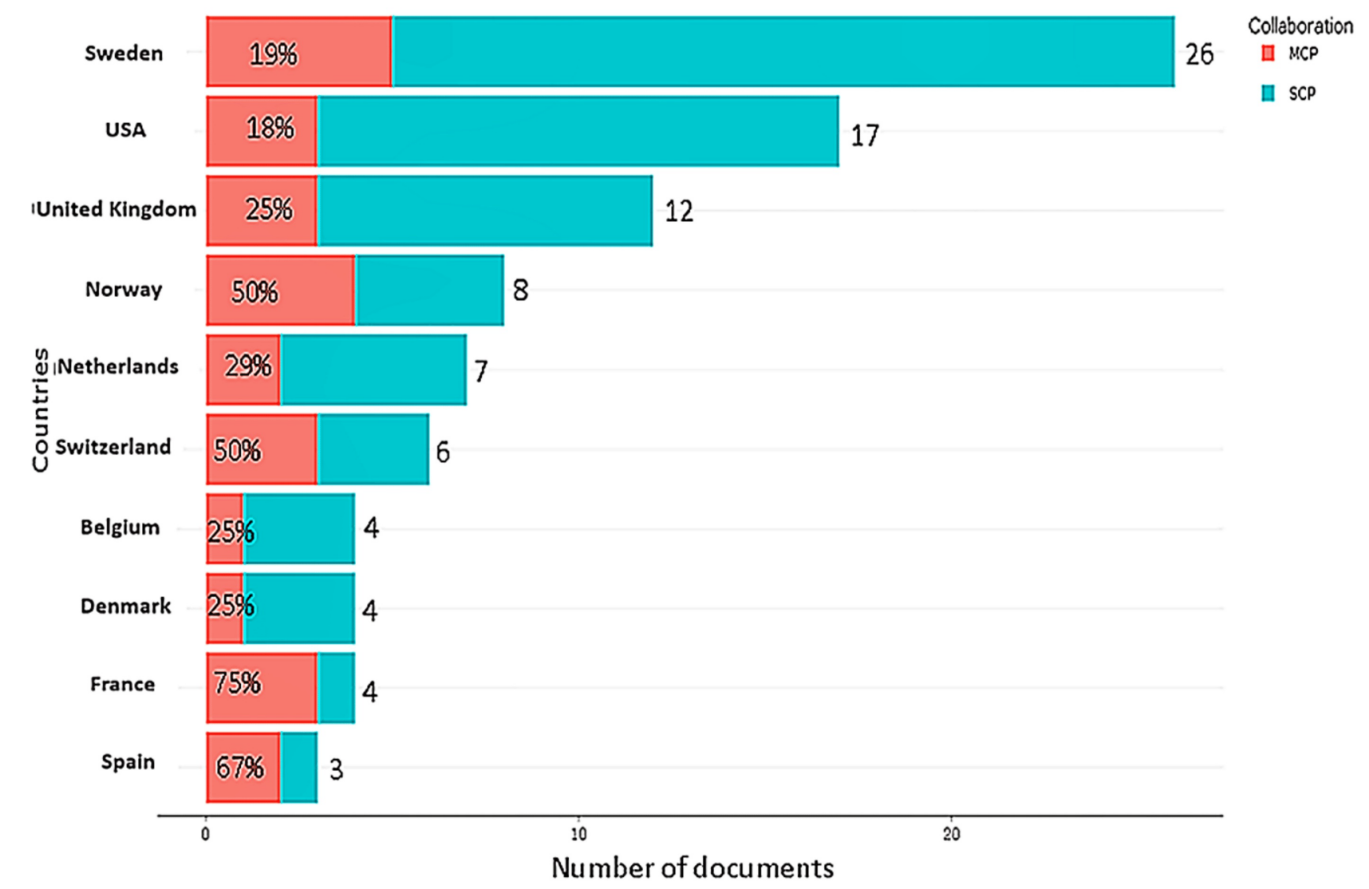
Top 100 most-cited articles on soccer injuries Top 10 corresponding authors’ countries of origin, along with Single Country Publications (SCP) and Multiple Country Publications (MCP).

Figure 2 shows the top ten corresponding authors’ countries of origin along with intra- and inter-countries collaboration. The top three countries were Sweden (n= 26 articles), USA (n= 17 articles), and United Kingdom (n= 12 articles).

**Figure 3:**
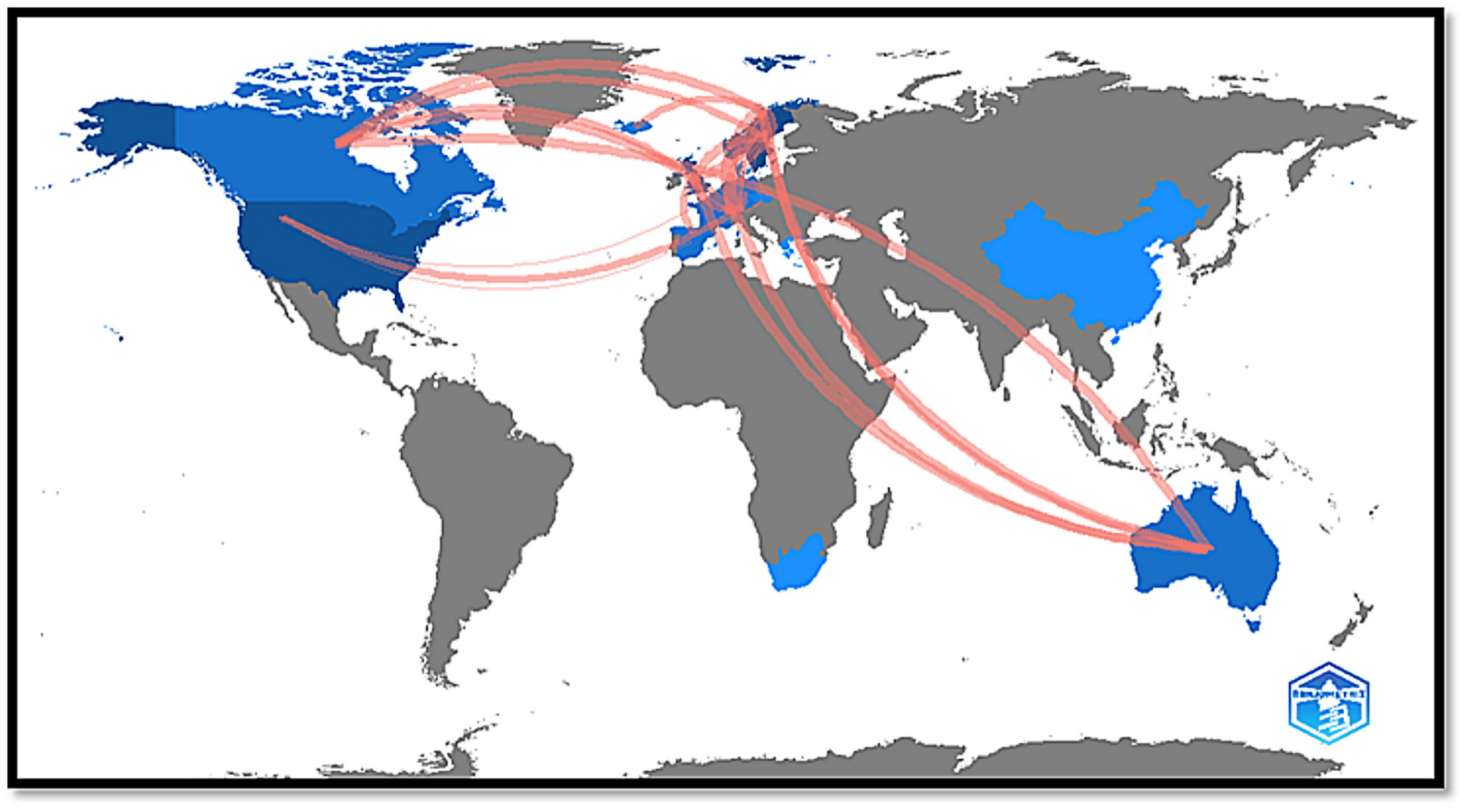
Top 100 most-cited articles on soccer injuries (inter-countries collaboration world map) The authors declare that Figure 2 was created using the Bibliometrix R-package software (https://www.bibliometrix.org/).

Figure 3 shows a world map of inter-country collaborations. These collaborations occurred especially between Norway, Sweden, and Switzerland.

**Figure 4:**
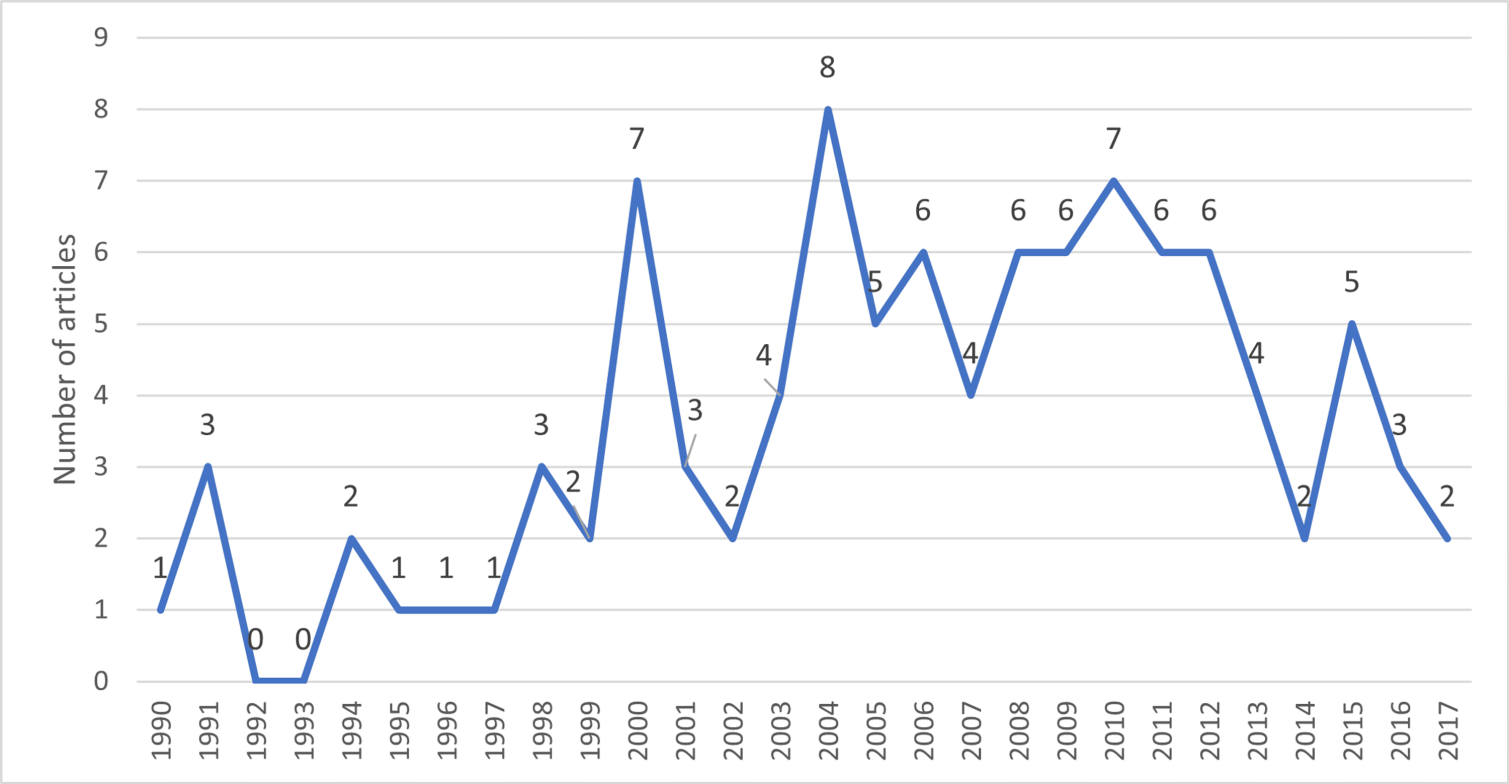
Top 100 most-cited articles on soccer injuries (annual publication of the selected articles)

Figure 4 exposes the annual production of the top 100 most-cited articles on soccer injuries. The latter were published between 1990 and 2017, with an average of 3.57 publications per year.

**Figure 5:**
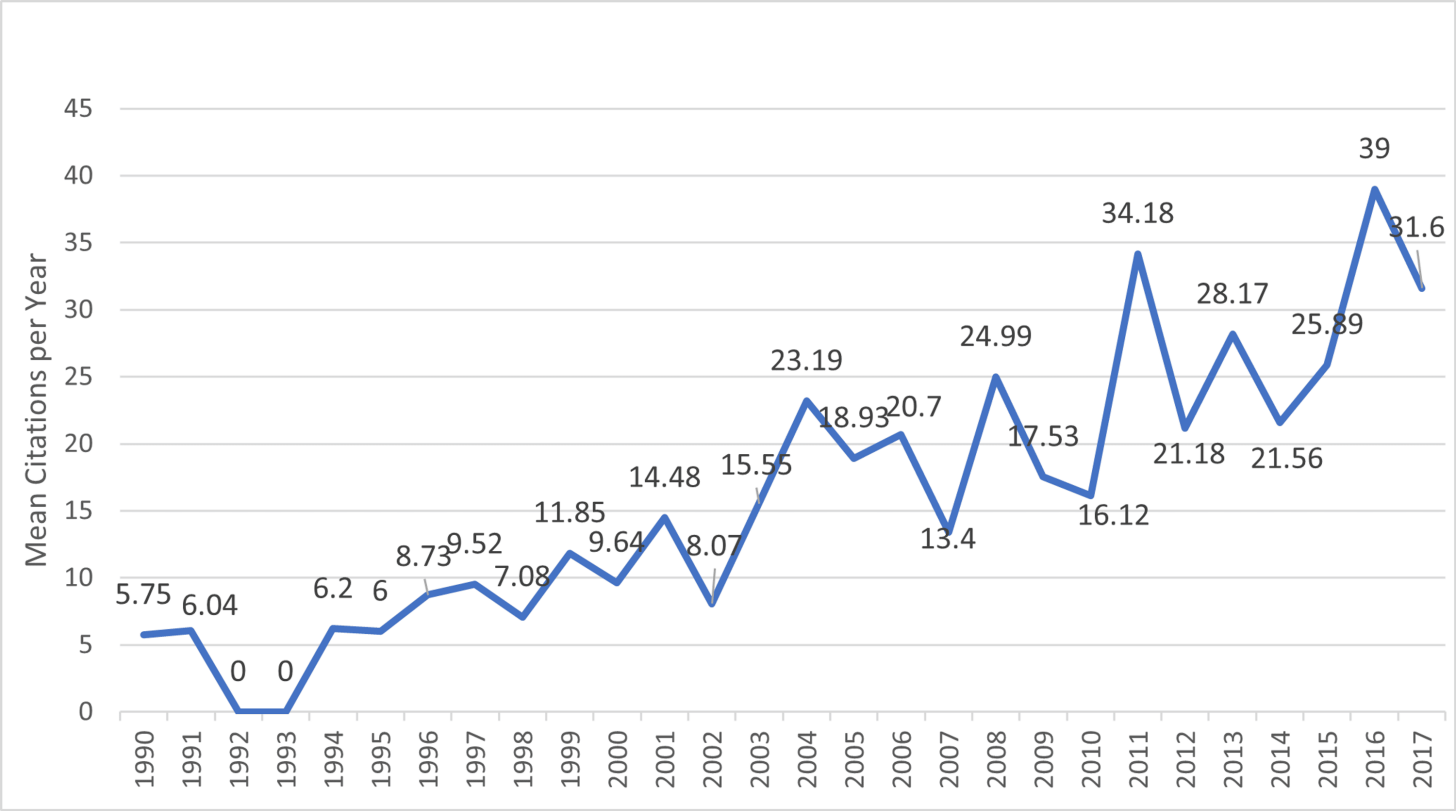
Top 100 most-cited articles on soccer injuries Mean number of citations per article per year is shown in the graph.

Figure 5 shows the mean number of citations per article and per year. The mean (range) number of citations per year and per article was 17.65 (5.75-39). The most recent article was published in April 2017 and was the only meta-analysis in the list, with a total 146 citations and 24.33 citations per year [16].

Table 3 exposes some characteristics of the top 100 most-cited articles on soccer injuries. First, the level of evidence was one in 20 articles, and two in 50 articles. Second, based on article type and study design, 73 were original research articles, and half were observational studies. Third, the most common research topics were epidemiology (n=26 articles), prevention (n=24 articles), and injury risk factors (n=20 articles). Finally, most of the top 100 most-cited articles were focused on males (n=53 articles), adults (n=57 articles), and elite soccer players (n=51 articles).

**Table 3:** Descriptive data of the top 100 most-cited articles relating to soccer injuries

Variable	Number of articles
Level of evidence	
1	20
2	50
3	26
4	3
5	1
Article type	
Original research	73
Review article	16
Case studies	9
Methodologies	1
Letter to the editor	1
Study design	
Observational	49
Descriptive	10
Experimental	24
Review	16
Methodology	1
Topic	
Epidemiology	26
Prevention	24
Injuries risk factors	20
Anatomy and biomechanics	11
Classification and scoring	7
Injuries mechanism	4
Rehabilitation	3
Imaging	2
Surgical technique and outcome	2
Neuropsychology	1
Sex	
Male	53
Female	13
Both	34
Age	
Youth	16
Adults	57
Both	27
Competition level	
Elite	51
Non-elite	17
Both	32

Among the 53 studies that included only male participants, 37 (69.8%) focused on elite players. Among the 13 studies that examined only female participants, eight (61.6%) involved non-elite players, while three (23%) and two (15.4%) focused on elite players and both categories, respectively. Additionally, 12 studies (92.3%) were published in the top five journals ranked by the number of articles (Table 1), and ten (76.9%) were among the 50 most cited articles.

Discussion

This bibliometric study aimed to identify the 100 most-cited articles related to soccer injuries and determine their characteristics to understand the research and publishing trends in this area. We found that the 100 most-cited articles were published between 1990 and 2017, mainly in the British Journal of Sports Medicine and the American Journal of Sports Medicine. In fact, the American Journal of Sports Medicine has shown a noticeable increase in the number of articles and citations in the past decades [17]. Furthermore, authors from high-ranking institutions who produce a high number of publications frequently publish in these journals. Thus, this may explain their high rate of citations [18,19]. Among the five most-cited authors, three (Hagglund M, Ekstrand J, and Walden M) are from Sweden and affiliated with the Linkoping University, and two (Jiri Dvorak (Dvorak J) and Astrid Junge (Jung A) are affiliated with the FIFA Medical Assessment and Research Centre in Zurich, Switzerland. These five most-cited authors each have more than 200 articles published in the field of sports sciences. Moreover, the three most-cited authors (Hagglund M, Ekstrand J, and Walden M) were involved in writing the two articles with the highest number of citations per year (60.75 and 55.33) [4,20].

The lack of publications and authors from Africa can be related to the paucity of surveillance facilities for soccer injuries in African competitions, such as FIFA excellence centres, which are fewer compared to Europe and North American countries [21]. Many African and South American countries belong to middle and lower-income countries, thus scientific research is often neglected in favor of meeting basic necessities. Therefore, the gap between African and South American countries and Western nations is made worse by the absence of investment in infrastructure and research funding. Moreover, structural barriers such as scarce opportunities for international collaboration and the inability to afford article processing charges (APCs), which are common in high-impact journals, further hinder research output from African countries [22].

Asian countries don’t have a great soccer tradition, but there is a relatively recent interest in soccer [23]. For example, Gulf nations such as the United Arab Emirates and Qatar have recently been actively establishing research institutions, augmenting allocations for research funds, and attracting international research talent to elevate their academic standing in sport science [24].

Original articles were the most common article type among the 100 most-cited articles, and half of them had an observational design. The main topics of the selected articles were epidemiology, prevention, and injury risk factors. The major challenge for research in sports, especially in soccer, remains injury prevention [23,25]. This may explain most authors’ interest in performing observational and descriptive studies. Epidemiology, risk factors, and prevention present the four steps of van Mechelen's sequence of prevention model [26] which is widely used to determine the extent of the injury; figuring out the mechanisms, risk factors, and injury causation; adopting preventive measures; and, at the end, assessing their efficacy. Remarkably, among the 100 most cited articles, only 24 were experimental studies. This is in concordance with the findings of Chalmers DJ, who explained the limited number of experimental studies, particularly randomized controlled trials, by the difficulties in accessing the population of interest and acquiring consistent measures of person-time exposure [27]. This shows that there's still much more need for studies in soccer medicine, particularly experimental ones, in order to better manage soccer injuries.

Despite the recent increase in the number of studies focusing on female players during the past two decades, fewer studies have focused on female players than male players [1]. However, articles about the female population seem highly citable. Globally, studies about elite athletes are cited more than those about recreational athletes. Their performance and financial imperatives may be the main explanation for this discrepancy [5,28].

Study limitations encompassed essentially the use of only the WoS database in our research. However, WoS was selected due to its stricter journal inclusion criteria, which ensure higher data quality. It also provides longer historical coverage, superior citation analysis tools, and better compatibility with bibliometric software. Moreover, WoS minimizes duplication and indexing inconsistencies, making it a more reliable source for identifying high-impact literature in orthopaedics and sports medicine. Also, the trends were based on authors’ keywords, which are sometimes chosen from predefined lists or free-text entries, and this could influence the words used as keywords. Furthermore, by focusing exclusively on the 100 most-cited articles, the study may not capture the full breadth and diversity of soccer injury research, particularly more recent or less-cited studies that may nonetheless offer valuable insights.

Our findings may help advance knowledge of soccer injuries and address a significant literature gap. In fact, researchers and sports medicine practitioners can make informed decisions, aligning their work with the results of influential publications. Additionally, the study identifies deficiencies in research, especially experimental trials, and orients further research.

## Conclusions

This bibliometric analysis provides valuable insights into the most influential research on soccer injuries, emphasizing the predominance of descriptive studies focused on epidemiology, risk factors, and prevention. While male professional players remain the primary study population, the high citation impact of articles on female players suggests a growing research interest in this area. Notably, the findings highlight a critical gap in experimental trials and a disparity in impactful research across different regions, with African and Asian studies being underrepresented. Addressing these deficiencies could enhance the global understanding of soccer-related injuries and promote more inclusive and regionally relevant research. By identifying key trends and research gaps, this study offers a roadmap for future investigations that could shape both academic inquiry and practical advancements in sports medicine.

## Appendices

**Table 4:** The top 100 most-cited articles on soccer injuries: rank, number of citations and citations per year

Rank	Paper	No. of citations	No. of citations/year
1	Lohmander LS, Ostenberg A, Englund M, Roos H. High prevalence of knee osteoarthritis, pain, and functional limitations in female soccer players twelve years after anterior cruciate ligament injury. Arthritis Rheum. 2004 Oct;50(10):3145-52.	945	49.74
2	Fuller CW, Ekstrand J, Junge A, Andersen TE, Bahr R, Dvorak J, Hägglund M, McCrory P, Meeuwisse WH. Consensus statement on injury definitions and data collection procedures in studies of football (soccer) injuries. Br J Sports Med. 2006 Mar;40(3):193-201.	794	46.71
3	Ekstrand J, Hägglund M, Waldén M. Injury incidence and injury patterns in professional football: the UEFA injury study. Br J Sports Med. 2011 Jun;45(7):553-8.	729	60.75
4	Ekstrand J, Hägglund M, Waldén M. Epidemiology of muscle injuries in professional football (soccer). Am J Sports Med. 2011 Jun;39(6):1226-32.	664	55.33
5	Mandelbaum BR, Silvers HJ, Watanabe DS, Knarr JF, Thomas SD, Griffin LY, Kirkendall DT, Garrett W Jr. Effectiveness of a neuromuscular and proprioceptive training program in preventing anterior cruciate ligament injuries in female athletes: 2-year follow-up. Am J Sports Med. 2005 Jul;33(7):1003-10.	664	36.89
6	Croisier JL, Ganteaume S, Binet J, Genty M, Ferret JM. Strength imbalances and prevention of hamstring injury in professional soccer players: a prospective study. Am J Sports Med. 2008 Aug;36(8):1469-75.	559	37.27
7	von Porat A, Roos EM, Roos H. High prevalence of osteoarthritis 14 years after an anterior cruciate ligament tear in male soccer players: a study of radiographic and patient relevant outcomes. Ann Rheum Dis. 2004 Mar;63(3):269-73.	557	29.32
8	Woods C, Hawkins RD, Maltby S, Hulse M, Thomas A, Hodson A; Football Association Medical Research Programme. The Football Association Medical Research Programme: an audit of injuries in professional football--analysis of hamstring injuries. Br J Sports Med. 2004 Feb;38(1):36-41.	555	29.21
9	Arnason A, Sigurdsson SB, Gudmundsson A, Holme I, Engebretsen L, Bahr R. Risk factors for injuries in football. Am J Sports Med. 2004 Jan-Feb;32(1 Suppl):5S-16S.	536	28.21
10	Alentorn-Geli E, Myer GD, Silvers HJ, Samitier G, Romero D, Lázaro-Haro C, Cugat R. Prevention of non-contact anterior cruciate ligament injuries in soccer players. Part 1: Mechanisms of injury and underlying risk factors. Knee Surg Sports Traumatol Arthrosc. 2009 Jul;17(7):705-29.	487	34.79
11	Hawkins RD, Hulse MA, Wilkinson C, Hodson A, Gibson M. The association football medical research programme: an audit of injuries in professional football. Br J Sports Med. 2001 Feb;35(1):43-7.	472	21.45
12	Askling C, Karlsson J, Thorstensson A. Hamstring injury occurrence in elite soccer players after preseason strength training with eccentric overload. Scand J Med Sci Sports. 2003 Aug;13(4):244-50.	469	23.45
13	Soligard T, Myklebust G, Steffen K, Holme I, Silvers H, Bizzini M, Junge A, Dvorak J, Bahr R, Andersen TE. Comprehensive warm-up programme to prevent injuries in young female footballers: cluster randomised controlled trial. BMJ. 2008 Dec 9;337:a2469.	445	29.67
14	Witvrouw E, Danneels L, Asselman P, D'Have T, Cambier D. Muscle flexibility as a risk factor for developing muscle injuries in male professional soccer players. A prospective study. Am J Sports Med. 2003 Jan-Feb;31(1):41-6.	399	19.95
15	Hägglund M, Waldén M, Ekstrand J. Previous injury as a risk factor for injury in elite football: a prospective study over two consecutive seasons. Br J Sports Med. 2006 Sep;40(9):767-72.	371	21.82
16	Hägglund M, Waldén M, Magnusson H, Kristenson K, Bengtsson H, Ekstrand J. Injuries affect team performance negatively in professional football: an 11-year follow-up of the UEFA Champions League injury study. Br J Sports Med. 2013 Aug;47(12):738-42.	370	37.00
17	Arnason A, Andersen TE, Holme I, Engebretsen L, Bahr R. Prevention of hamstring strains in elite soccer: an intervention study. Scand J Med Sci Sports. 2008 Feb;18(1):40-8.	349	23.27
18	Petersen J, Thorborg K, Nielsen MB, Budtz-Jørgensen E, Hölmich P. Preventive effect of eccentric training on acute hamstring injuries in men's soccer: a cluster-randomized controlled trial. Am J Sports Med. 2011 Nov;39(11):2296-303.	344	28.67
19	Hägglund M, Waldén M, Bahr R, Ekstrand J. Methods for epidemiological study of injuries to professional football players: developing the UEFA model. Br J Sports Med. 2005 Jun;39(6):340-6.	341	18.94
20	Gilchrist J, Mandelbaum BR, Melancon H, Ryan GW, Silvers HJ, Griffin LY, Watanabe DS, Dick RW, Dvorak J. A randomized controlled trial to prevent noncontact anterior cruciate ligament injury in female collegiate soccer players. Am J Sports Med. 2008 Aug;36(8):1476-83.	339	22.60
21	Ekstrand J, Waldén M, Hägglund M. Hamstring injuries have increased by 4% annually in men's professional football, since 2001: a 13-year longitudinal analysis of the UEFA Elite Club injury study. Br J Sports Med. 2016 Jun;50(12):731-7.	330	47.14
22	Heidt RS Jr, Sweeterman LM, Carlonas RL, Traub JA, Tekulve FX. Avoidance of soccer injuries with preseason conditioning. Am J Sports Med. 2000 Sep-Oct;28(5):659-62.	285	12.39
23	Hölmich P, Uhrskou P, Ulnits L, Kanstrup IL, Nielsen MB, Bjerg AM, Krogsgaard K. Effectiveness of active physical training as treatment for long-standing adductor-related groin pain in athletes: randomised trial. Lancet. 1999 Feb 6;353(9151):439-43.	273	11.38
24	Matser EJ, Kessels AG, Lezak MD, Jordan BD, Troost J. Neuropsychological impairment in amateur soccer players. JAMA. 1999 Sep 8;282(10):971-3.	272	11.33
25	Waldén M, Hägglund M, Ekstrand J. UEFA Champions League study: a prospective study of injuries in professional football during the 2001-2002 season. Br J Sports Med. 2005 Aug;39(8):542-6.	267	14.83
26	Ekstrand J, Healy JC, Waldén M, Lee JC, English B, Hägglund M. Hamstring muscle injuries in professional football: the correlation of MRI findings with return to play. Br J Sports Med. 2012 Feb;46(2):112-7.	259	23.55
27	Dupont G, Nedelec M, McCall A, McCormack D, Berthoin S, Wisløff U. Effect of 2 soccer matches in a week on physical performance and injury rate. Am J Sports Med. 2010 Sep;38(9):1752-8.	257	19.77
28	Yu B, Garrett WE. Mechanisms of non-contact ACL injuries. Br J Sports Med. 2007 Aug;41 Suppl 1(Suppl 1):i47-51.	254	15.88
29	Fuller CW, Ekstrand J, Junge A, Andersen TE, Bahr R, Dvorak J, Hägglund M, McCrory P, Meeuwisse WH. Consensus statement on injury definitions and data collection procedures in studies of football (soccer) injuries. Scand J Med Sci Sports. 2006 Apr;16(2):83-92.	249	14.65
30	Fuller CW, Ekstrand J, Junge A, Andersen TE, Bahr R, Dvorak J, Hägglund M, McCrory P, Meeuwisse WH. Consensus statement on injury definitions and data collection procedures in studies of football (soccer) injuries. Clin J Sport Med. 2006 Mar;16(2):97-106.	246	14.47
31	Heiderscheit BC, Sherry MA, Silder A, Chumanov ES, Thelen DG. Hamstring strain injuries: recommendations for diagnosis, rehabilitation, and injury prevention. J Orthop Sports Phys Ther. 2010 Feb;40(2):67-81.	244	18.77
32	Söderman K, Werner S, Pietilä T, Engström B, Alfredson H. Balance board training: prevention of traumatic injuries of the lower extremities in female soccer players? A prospective randomized intervention study. Knee Surg Sports Traumatol Arthrosc. 2000;8(6):356-63.	239	10.39
33	Nédélec M, McCall A, Carling C, Legall F, Berthoin S, Dupont G. Recovery in soccer: part I - post-match fatigue and time course of recovery. Sports Med. 2012 Dec 1;42(12):997-1015.	238	21.64
34	Bjordal JM, Arnły F, Hannestad B, Strand T. Epidemiology of anterior cruciate ligament injuries in soccer. Am J Sports Med. 1997 May-Jun;25(3):341-5.	238	9.15
35	Steffen K, Myklebust G, Olsen OE, Holme I, Bahr R. Preventing injuries in female youth football--a cluster-randomized controlled trial. Scand J Med Sci Sports. 2008 Oct;18(5):605-14.	230	15.33
36	Söderman K, Alfredson H, Pietilä T, Werner S. Risk factors for leg injuries in female soccer players: a prospective investigation during one out-door season. Knee Surg Sports Traumatol Arthrosc. 2001 Sep;9(5):313-21.	230	10.45
37	Waldén M, Atroshi I, Magnusson H, Wagner P, Hägglund M. Prevention of acute knee injuries in adolescent female football players: cluster randomised controlled trial. BMJ. 2012 May 3;344:e3042.	228	20.73
38	Peterson L, Junge A, Chomiak J, Graf-Baumann T, Dvorak J. Incidence of football injuries and complaints in different age groups and skill-level groups. Am J Sports Med. 2000;28(5 Suppl):S51-7.	228	9.91
39	Arnason A, Gudmundsson A, Dahl HA, Jóhannsson E. Soccer injuries in Iceland. Scand J Med Sci Sports. 1996 Feb;6(1):40-5.	227	8.41
40	Lipton ML, Kim N, Zimmerman ME, Kim M, Stewart WF, Branch CA, Lipton RB. Soccer heading is associated with white matter microstructural and cognitive abnormalities. Radiology. 2013 Sep;268(3):850-7.	226	22.60
41	Colvin AC, Mullen J, Lovell MR, West RV, Collins MW, Groh M. The role of concussion history and gender in recovery from soccer-related concussion. Am J Sports Med. 2009 Sep;37(9):1699-704.	226	16.14
42	Boytim MJ, Fischer DA, Neumann L. Syndesmotic ankle sprains. Am J Sports Med. 1991 May-Jun;19(3):294-8.	225	7.03
43	Matser JT, Kessels AG, Jordan BD, Lezak MD, Troost J. Chronic traumatic brain injury in professional soccer players. Neurology. 1998 Sep;51(3):791-6.	216	8.64
44	Brophy RH, Schmitz L, Wright RW, Dunn WR, Parker RD, Andrish JT, McCarty EC, Spindler KP. Return to play and future ACL injury risk after ACL reconstruction in soccer athletes from the Multicenter Orthopaedic Outcomes Network (MOON) group. Am J Sports Med. 2012 Nov;40(11):2517-22.	215	19.55
45	Timmins RG, Bourne MN, Shield AJ, Williams MD, Lorenzen C, Opar DA. Short biceps femoris fascicles and eccentric knee flexor weakness increase the risk of hamstring injury in elite football (soccer): a prospective cohort study. Br J Sports Med. 2016 Dec;50(24):1524-1535.	213	30.43
46	Steffen K, Emery CA, Romiti M, Kang J, Bizzini M, Dvorak J, Finch CF, Meeuwisse WH. High adherence to a neuromuscular injury prevention programme (FIFA 11+) improves functional balance and reduces injury risk in Canadian youth female football players: a cluster randomised trial. Br J Sports Med. 2013 Aug;47(12):794-802.	212	21.20
47	Agel J, Evans TA, Dick R, Putukian M, Marshall SW. Descriptive epidemiology of collegiate men's soccer injuries: National Collegiate Athletic Association Injury Surveillance System, 1988-1989 through 2002-2003. J Athl Train. 2007 Apr-Jun;42(2):270-7.	210	13.13
48	Price RJ, Hawkins RD, Hulse MA, Hodson A. The Football Association medical research programme: an audit of injuries in academy youth football. Br J Sports Med. 2004 Aug;38(4):466-71.	210	11.05
49	Drawer S, Fuller CW. Propensity for osteoarthritis and lower limb joint pain in retired professional soccer players. Br J Sports Med. 2001 Dec;35(6):402-8.	210	9.55
50	Padua DA, DiStefano LJ, Beutler AI, de la Motte SJ, DiStefano MJ, Marshall SW. The Landing Error Scoring System as a Screening Tool for an Anterior Cruciate Ligament Injury-Prevention Program in Elite-Youth Soccer Athletes. J Athl Train. 2015 Jun;50(6):589-95.	207	25.88
51	Hägglund M, Waldén M, Ekstrand J. Risk factors for lower extremity muscle injury in professional soccer: the UEFA Injury Study. Am J Sports Med. 2013 Feb;41(2):327-35.	206	20.60
52	van der Horst N, Smits DW, Petersen J, Goedhart EA, Backx FJ. The preventive effect of the nordic hamstring exercise on hamstring injuries in amateur soccer players: a randomized controlled trial. Am J Sports Med. 2015 Jun;43(6):1316-23.	205	25.63
53	Croisier JL. Factors associated with recurrent hamstring injuries. Sports Med. 2004;34(10):681-95.	198	10.42
54	Surve I, Schwellnus MP, Noakes T, Lombard C. A fivefold reduction in the incidence of recurrent ankle sprains in soccer players using the Sport-Stirrup orthosis. Am J Sports Med. 1994 Sep-Oct;22(5):601-6.	198	6.83
55	Dvorak J, Junge A. Football injuries and physical symptoms. A review of the literature. Am J Sports Med. 2000;28(5 Suppl):S3-9.	196	8.52
56	Dick R, Putukian M, Agel J, Evans TA, Marshall SW. Descriptive epidemiology of collegiate women's soccer injuries: National Collegiate Athletic Association Injury Surveillance System, 1988-1989 through 2002-2003. J Athl Train. 2007 Apr-Jun;42(2):278-85.	193	12.06
57	Ostenberg A, Roos H. Injury risk factors in female European football. A prospective study of 123 players during one season. Scand J Med Sci Sports. 2000 Oct;10(5):279-85.	192	8.35
58	Fousekis K, Tsepis E, Poulmedis P, Athanasopoulos S, Vagenas G. Intrinsic risk factors of non-contact quadriceps and hamstring strains in soccer: a prospective study of 100 professional players. Br J Sports Med. 2011 Jul;45(9):709-14.	189	15.75
59	Shea KG, Pfeiffer R, Wang JH, Curtin M, Apel PJ. Anterior cruciate ligament injury in pediatric and adolescent soccer players: an analysis of insurance data. J Pediatr Orthop. 2004 Nov-Dec;24(6):623-8	189	9.95
60	Waldén M, Krosshaug T, Bjørneboe J, Andersen TE, Faul O, Hägglund M. Three distinct mechanisms predominate in non-contact anterior cruciate ligament injuries in male professional football players: a systematic video analysis of 39 cases. Br J Sports Med. 2015 Nov;49(22):1452-60	188	23.50
61	Alentorn-Geli E, Myer GD, Silvers HJ, Samitier G, Romero D, Lázaro-Haro C, Cugat R. Prevention of non-contact anterior cruciate ligament injuries in soccer players. Part 2: a review of prevention programs aimed to modify risk factors and to reduce injury rates. Knee Surg Sports Traumatol Arthrosc. 2009 Aug;17(8):859-79.	185	13.21
62	Ekstrand J, Tropp H. The incidence of ankle sprains in soccer. Foot Ankle. 1990 Aug;11(1):41-4.	184	5.58
63	Soligard T, Nilstad A, Steffen K, Myklebust G, Holme I, Dvorak J, Bahr R, Andersen TE. Compliance with a comprehensive warm-up programme to prevent injuries in youth football. Br J Sports Med. 2010 Sep;44(11):787-93.	182	14.00
64	Chomiak J, Junge A, Peterson L, Dvorak J. Severe injuries in football players. Influencing factors. Am J Sports Med. 2000;28(5 Suppl):S58-68.	182	7.91
65	Engström B, Johansson C, Törnkvist H. Soccer injuries among elite female players. Am J Sports Med. 1991 Jul-Aug;19(4):372-5.	180	5.63
66	Agricola R, Heijboer MP, Ginai AZ, Roels P, Zadpoor AA, Verhaar JA, Weinans H, Waarsing JH. A cam deformity is gradually acquired during skeletal maturation in adolescent and young male soccer players: a prospective study with minimum 2-year follow-up. Am J Sports Med. 2014 Apr;42(4):798-806.	178	19.78
67	Engebretsen AH, Myklebust G, Holme I, Engebretsen L, Bahr R. Prevention of injuries among male soccer players: a prospective, randomized intervention study targeting players with previous injuries or reduced function. Am J Sports Med. 2008 Jun;36(6):1052-60.	177	11.80
68	Wong P, Hong Y. Soccer injury in the lower extremities. Br J Sports Med. 2005 Aug;39(8):473-82.	176	9.78
69	Emery CA, Meeuwisse WH. The effectiveness of a neuromuscular prevention strategy to reduce injuries in youth soccer: a cluster-randomised controlled trial. Br J Sports Med. 2010 Jun;44(8):555-62.	175	13.46
70	Waldén M, Hägglund M, Magnusson H, Ekstrand J. Anterior cruciate ligament injury in elite football: a prospective three-cohort study. Knee Surg Sports Traumatol Arthrosc. 2011 Jan;19(1):11-9.	173	14.42
71	Brophy R, Silvers HJ, Gonzales T, Mandelbaum BR. Gender influences: the role of leg dominance in ACL injury among soccer players. Br J Sports Med. 2010 Aug;44(10):694-7.	172	13.23
72	Le Gall F, Carling C, Reilly T, Vandewalle H, Church J, Rochcongar P. Incidence of injuries in elite French youth soccer players: a 10-season study. Am J Sports Med. 2006 Jun;34(6):928-38.	172	10.12
73	Woods C, Hawkins R, Hulse M, Hodson A. The Football Association Medical Research Programme: an audit of injuries in professional football-analysis of preseason injuries. Br J Sports Med. 2002 Dec;36(6):436-41; discussion 441.	171	8.14
74	Bowen L, Gross AS, Gimpel M, Li FX. Accumulated workloads and the acute:chronic workload ratio relate to injury risk in elite youth football players. Br J Sports Med. 2017 Mar;51(5):452-459.	170	28.33
75	Agricola R, Bessems JH, Ginai AZ, Heijboer MP, van der Heijden RA, Verhaar JA, Weinans H, Waarsing JH. The development of Cam-type deformity in adolescent and young male soccer players. Am J Sports Med. 2012 May;40(5):1099-106.	170	15.45
76	McCall A, Carling C, Nedelec M, Davison M, Le Gall F, Berthoin S, Dupont G. Risk factors, testing and preventative strategies for non-contact injuries in professional football: current perceptions and practices of 44 teams from various premier leagues. Br J Sports Med. 2014 Sep;48(18):1352-7.	167	18.56
77	Brink MS, Visscher C, Arends S, Zwerver J, Post WJ, Lemmink KA. Monitoring stress and recovery: new insights for the prevention of injuries and illnesses in elite youth soccer players. Br J Sports Med. 2010 Sep;44(11):809-15.	165	12.69
78	Hägglund M, Waldén M, Ekstrand J. Injuries among male and female elite football players. Scand J Med Sci Sports. 2009 Dec;19(6):819-27.	165	11.79
79	Junge A, Dvorak J. Influence of definition and data collection on the incidence of injuries in football. Am J Sports Med. 2000;28(5 Suppl):S40-6.	163	7.09
80	Roos H, Ornell M, Gärdsell P, Lohmander LS, Lindstrand A. Soccer after anterior cruciate ligament injury--an incompatible combination? A national survey of incidence and risk factors and a 7-year follow-up of 310 players. Acta Orthop Scand. 1995 Apr;66(2):107-12.	162	5.79
81	Faude O, Junge A, Kindermann W, Dvorak J. Injuries in female soccer players: a prospective study in the German national league. Am J Sports Med. 2005 Nov;33(11):1694-700.	161	8.94
82	Koerte IK, Ertl-Wagner B, Reiser M, Zafonte R, Shenton ME. White matter integrity in the brains of professional soccer players without a symptomatic concussion. JAMA. 2012 Nov 14;308(18):1859-61.	161	14.64
83	Woods C, Hawkins R, Hulse M, Hodson A. The Football Association Medical Research Programme: an audit of injuries in professional football: an analysis of ankle sprains. Br J Sports Med. 2003 Jun;37(3):233-8.	160	8.00
84	Waldén M, Hägglund M, Magnusson H, Ekstrand J. ACL injuries in men's professional football: a 15-year prospective study on time trends and return-to-play rates reveals only 65% of players still play at the top level 3 years after ACL rupture. Br J Sports Med. 2016 Jun;50(12):744-50.	159	22.71
85	Small K, McNaughton L, Greig M, Lovell R. The effects of multidirectional soccer-specific fatigue on markers of hamstring injury risk. J Sci Med Sport. 2010 Jan;13(1):120-5.	159	12.23
86	Waldén M, Hägglund M, Werner J, Ekstrand J. The epidemiology of anterior cruciate ligament injury in football (soccer): a review of the literature from a gender-related perspective. Knee Surg Sports Traumatol Arthrosc. 2011 Jan;19(1):3-10.	157	13.08
87	Werner J, Hägglund M, Waldén M, Ekstrand J. UEFA injury study: a prospective study of hip and groin injuries in professional football over seven consecutive seasons. Br J Sports Med. 2009 Dec;43(13):1036-40.	157	11.21
88	Tysvaer AT, Løchen EA. Soccer injuries to the brain. A neuropsychologic study of former soccer players. Am J Sports Med. 1991 Jan-Feb;19(1):56-60.	157	4.91
89	Ekstrand J, Timpka T, Hägglund M. Risk of injury in elite football played on artificial turf versus natural grass: a prospective two-cohort study. Br J Sports Med. 2006 Dec;40(12):975-80.	155	9.12
90	Bizzini M, Dvorak J. FIFA 11+: an effective programme to prevent football injuries in various player groups worldwide-a narrative review. Br J Sports Med. 2015 May;49(9):577-9.	154	19.25
91	Sánchez M, Azofra J, Anitua E, Andía I, Padilla S, Santisteban J, Mujika I. Plasma rich in growth factors to treat an articular cartilage avulsion: a case report. Med Sci Sports Exerc. 2003 Oct;35(10):1648-52.	154	7.70
92	Silvers-Granelli H, Mandelbaum B, Adeniji O, Insler S, Bizzini M, Pohlig R, Junge A, Snyder-Mackler L, Dvorak J. Efficacy of the FIFA 11+ Injury Prevention Program in the Collegiate Male Soccer Player. Am J Sports Med. 2015 Nov;43(11):2628-37.	152	19.00
93	Rahnama N, Reilly T, Lees A. Injury risk associated with playing actions during competitive soccer. Br J Sports Med. 2002 Oct;36(5):354-9.	152	7.24
94	Junge A, Dvorak J, Graf-Baumann T, Peterson L. Football injuries during FIFA tournaments and the Olympic Games, 1998-2001: development and implementation of an injury-reporting system. Am J Sports Med. 2004 Jan-Feb;32(1 Suppl):80S-9S.	149	7.84
95	Inklaar H. Soccer injuries. I: Incidence and severity. Sports Med. 1994 Jul;18(1):55-73.	149	5.14
96	Barnes BC, Cooper L, Kirkendall DT, McDermott TP, Jordan BD, Garrett WE Jr. Concussion history in elite male and female soccer players. Am J Sports Med. 1998 May-Jun;26(3):433-8.	148	5.92
97	Lehance C, Binet J, Bury T, Croisier JL. Muscular strength, functional performances and injury risk in professional and junior elite soccer players. Scand J Med Sci Sports. 2009 Apr;19(2):243-51.	147	10.50
98	Langberg H, Ellingsgaard H, Madsen T, Jansson J, Magnusson SP, Aagaard P, Kjaer M. Eccentric rehabilitation exercise increases peritendinous type I collagen synthesis in humans with Achilles tendinosis. Scand J Med Sci Sports. 2007 Feb;17(1):61-6.	147	9.19
99	Thorborg K, Krommes KK, Esteve E, Clausen MB, Bartels EM, Rathleff MS. Effect of specific exercise-based football injury prevention programmes on the overall injury rate in football: a systematic review and meta-analysis of the FIFA 11 and 11+ programmes. Br J Sports Med. 2017 Apr;51(7):562-571.	146	24.33
100	Boden BP, Kirkendall DT, Garrett WE Jr. Concussion incidence in elite college soccer players. Am J Sports Med. 1998 Mar-Apr;26(2):238-41.	146	5.84
